# 24-Methyl-Cholesta-5,24(28)-Diene-3β,19-diol-7β-Monoacetate Inhibits Human Small Cell Lung Cancer Growth In Vitro and In Vivo via Apoptosis Induction

**DOI:** 10.3390/md15070210

**Published:** 2017-07-01

**Authors:** Ting-Wen Chung, Jui-Hsin Su, Chi-Chen Lin, Yi-Rong Li, Ya-Hsuan Chao, Sheng-Hao Lin, Hong-Lin Chan

**Affiliations:** 1 Department of Medical Sciences, Institute of Bioinformatics and Structural Biology, National Tsing Hua University, Hsinchu 300, Taiwan; bominco@gmail.com; 2 Taiwan Coral Research Center, National Museum of Marine Biology & Aquarium, Pingtung 944, Taiwan; x2219@nmmba.gov.tw; 3 Department of Life Sciences, Institute of Biomedical Science, National Chung Hsing University, Taichung 402, Taiwan; lincc@dragon.nchu.edu.tw (C.-C.L.); peanutsnoopyemmali@gmail.com (Y.-R.L.); demonsandy@gmail.com (Y.-H.C.); 4 Department of Internal Medicine, Changhua Christian Hospital, Changhua Division of Chest Medicine, Changhua 500, Taiwan

**Keywords:** 24-methyl-cholesta-5,24(28)-diene-3β,19-diol-7β-monoacetate, MeCDDA, *Nephthea erecta*, small cell lung cancer, apoptosis

## Abstract

24-methyl-cholesta-5,24(28)-diene-3β,19-diol-7β-monoacetate (MeCDDA) is a natural steroid compound isolated from a wild-type soft coral (*Nephthea erecta*). The present study aimed to investigate the anti-small cell lung cancer (SCLC) effects of MeCDDA in vitro and in vivo, as well as to elucidate its underlying mechanism. Our results indicated that H1688 and H146 cells show relevant sensitivity to MeCDDA, and the exposure to MeCDDA in SCLC cells caused dose-dependent growth inhibitory responses. In addition, MeCDDA treatment promoted cell apoptosis and increased the activities of caspases in H1688 cells, reducing the mitochondrial membrane potential and stimulating the release of cytochrome c into the cytosol. Along with the increase in Bax expression and reduction in Bcl-2, the MeCDDA treatment also significantly decreased Akt and mTOR phosphorylation. Finally, MeCDDA treatment in the mouse xenograft model of H1688 cells exhibited significant inhibition of tumor growth, corroborating MeCDDA as a potential pre-clinical candidate for the treatment of SCLC. Overall, our results demonstrate that the cytotoxic effects of MeCDDA towards H1688 and H146 cells, possibly through the activation of the mitochondrial apoptotic pathway and inhibition of the PI3K/Akt/mTOR pathway, merit further studies for its possible clinical application in chemotherapy.

## 1. Introduction

Lung cancer is the most common cancer worldwide. In addition to a high incidence, lung cancer has the highest death rate among all cancer types [1]. Lung cancer can be divided into small cell lung cancer (SCLC) and non-small cell lung cancer (NSCLC) primarily based on specific histopathological features of the disease as well as other classification criteria, such as smoking exposure [2] and neuroendocrine properties. For example, the l-dopa decarboxylase activity is higher in SCLC specimens but not in NSCLC [3]. The distinction between SCLC and NSCLC is important for treating SCLC patients because SCLC appears to show better chemosensitivity than NSCLC patients [4]. In general, SCLC accounts for approximately 10% to 15% of all lung cancers [5]. Clinically, SCLC is distinguished from NSCLC by high growth fraction, rapid tumor doubling time, and widespread metastasis [6]. According to the guidelines of the American Cancer Society [7], chemotherapy is typically part of the treatment for SCLC, and cisplatin, etoposide, carboplatin, and irinotecan are the most often-used drugs. However, the efficacy of current SCLC chemotherapy is modest [8], and SCLC treatment remains an unmet medical need.

Recently, natural products derived from marine organisms have been recognized as potential sources of valuable anti-cancer drug candidates in the biotechnology and pharmaceutical industries. Steroids are some of the commonly-encountered natural products in soft corals of the genus *Nephthea* [9]. Previous studies showed that they have a broad range of biological properties, such as anti-inflammatory [10], anti-fouling [11], and anti-cancer [10,12,13] effects.

24-methyl-cholesta-5,24(28)-diene-3β,19-diol-7β-monoacetate (MeCDDA) is a natural steroidal compound isolated from the cultured soft coral *Nephthea erecta* and has been found to exhibit cytotoxic effects against different types of human cancer cells [13], including human chronic myelogenous leukemia, human acute lymphoblastic leukemia, human T lymphoblastoid, and human leukemic monocyte lymphoma.

Although these findings demonstrate the anti-cancer activity of MeCDDA, the molecular and cellular mechanisms underlying its anti-cancer activities are not known and require further investigation. In addition, to date, there has been no study of the anti-tumor effects of MeCDDA against human SCLC cancer cells. Thus, in this study, we examined the cytotoxic effects of MeCDDA on human H1688 SCLC cells and studied the mechanisms underlying the induced cancer cell death.

## 2. Results

### 2.1. MeCDDA Decreased the Cell Viability of H1688 and H146 in vitro

**Figure 1:**
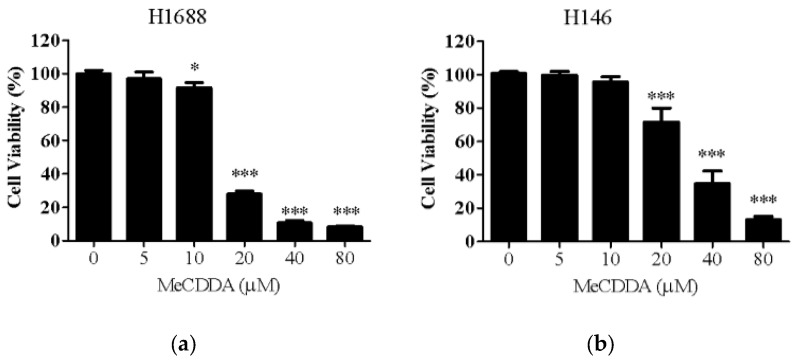
The viability of (**a**) H1688 and (**b**) H146 human SCLC cells was inhibited in a dose-dependent manner by treatment with 5–80 µM MeCDDA for 24 h. Cell growth was assessed by the MTT assay. Data are expressed as the percentage of the control treated with DMSO. Values represent means ± SD from triplicate samples for each treatment, * *p* < 0.05, *** *p* < 0.001 compared with control (one-way ANOVA).

First, we examined the effects of MeCDDA treatment on the cell viability of human small lung cancer cell lines, H1688 and H146, measured by MTT assay. As shown in Figure 1a, concentrations of MeCDDA higher than 20 µM exhibited significant cytotoxicity towards H1688 cells, and MeCDDA treatments on H146 SCLC cells showed similar, but relatively mild, cytotoxic effects (Figure 1b) after 24 h exposure. H1688 cells were more sensitive than H146 cells to MeCDDA. Therefore, subsequent experiments were conducted with H1688 cells.

### 2.2. MeCDDA Induced Apoptosis of H1688

**Figure 2:**
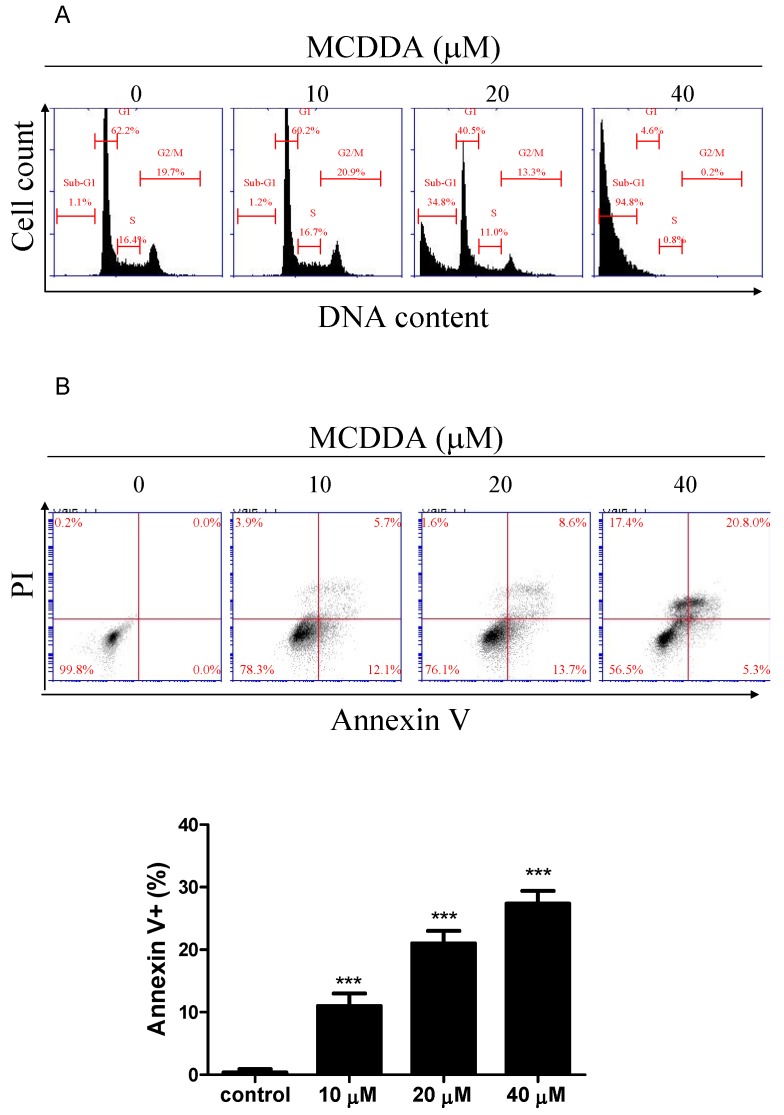
MeCDDA induced apoptosis of H1688 SCLC cells. (**A**) Detection of H1688 apoptotic cells after MeCDDA treatment by propidium iodide (PI) staining and subsequent flow cytometric analysis. Data are representative of three independent experiments showing similar results. (**B**) Detection of H1688 apoptotic cells after MeCDDA treatment by labeling for phosphatidylserine externalization with FITC-annexin-V and cell membrane integrity with PI. The cells were treated with the indicated concentrations of MeCDDA or DMSO for 24 h. Cells were harvested for PI and annexin V/PI staining as described in the Materials and Methods section. The mean ± SD of the experimental triplicates is presented in the bar graph, *** *p* < 0.001 compared with control (one-way ANOVA).

We observed that MeCDDA caused significant cytotoxicity in SCLC cells, so we wondered whether the decreased cell viability was associated with apoptosis. To this end, we performed flow cytometric analysis to measure the levels of apoptosis after MeCDDA treatment. Figure 2A and Table 1 show that MeCDDA induced an increase in cell sub-G_1_ population, which is an indication of cell death, and this effect was dose-dependent. In addition, to clarify the type of cell death elicited by MeCDDA, cells were subjected to flow cytometry analysis after staining with annexin V-FITC and propidium iodide (PI). As shown in Figure 2B, the percentages of early apoptotic death (annexin V^+^/PI^−^) and late apoptotic death (annexin V^+^/PI^+^) increased in a dose-dependent manner in H1688 cells. Taken together, these results suggest that MeCDDA induces apoptosis in H1688 cells.

### 2.3. MeCDDA Induced H1688 Cells Apoptosis through a Caspase-Dependent Pathway

**Figure 3:**
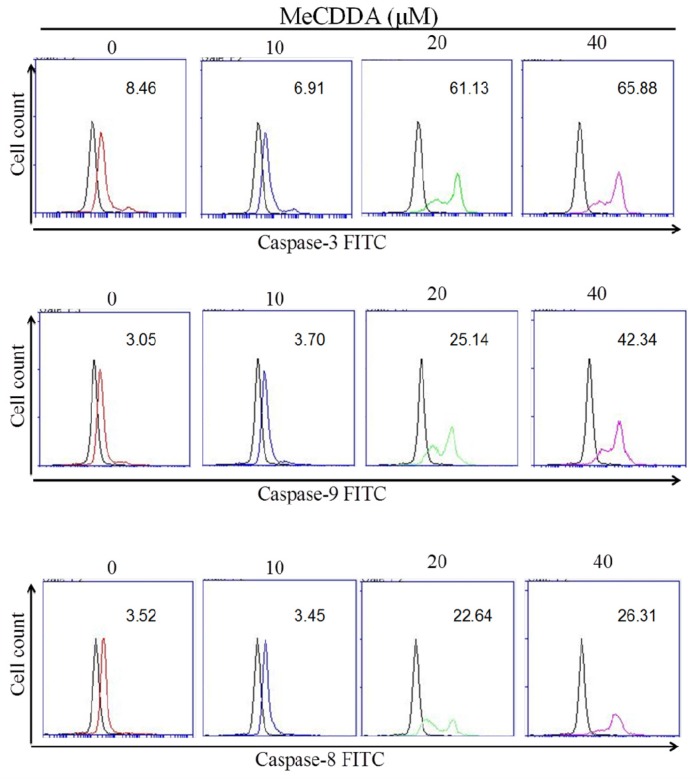
MeCDDA induced caspase-3, caspase-8, and caspase-9 activity in H1688 cells. H1688 cells were treated with MeCDDA or DMSO for 24 h and then the activities of caspase-3, -8, and -9 were determined by flow cytometry. Black line: unstained H1688 cell control; red line: mock control of DMSO-treated cell control; blue line: cells treated with 10 µM MeCDDA; green line: cells treated with 20 µM MeCDDA; violet line: cells treated with 40 µM MeCDDA. Data are representative of three independent experiments showing similar results.

After treatment with MeCDDA for 24 h, activities of caspase-3, caspase-8, and caspase-9 were examined (Figure 3). The results indicate that MeCDDA significantly increased caspase-3, caspase-8, and caspase-9 activities in H1688 cells.

### 2.4. DA-Induced Loss of Mitochondrial Membrane Potential and Facilitated Cytochrome C Release

**Figure 4:**
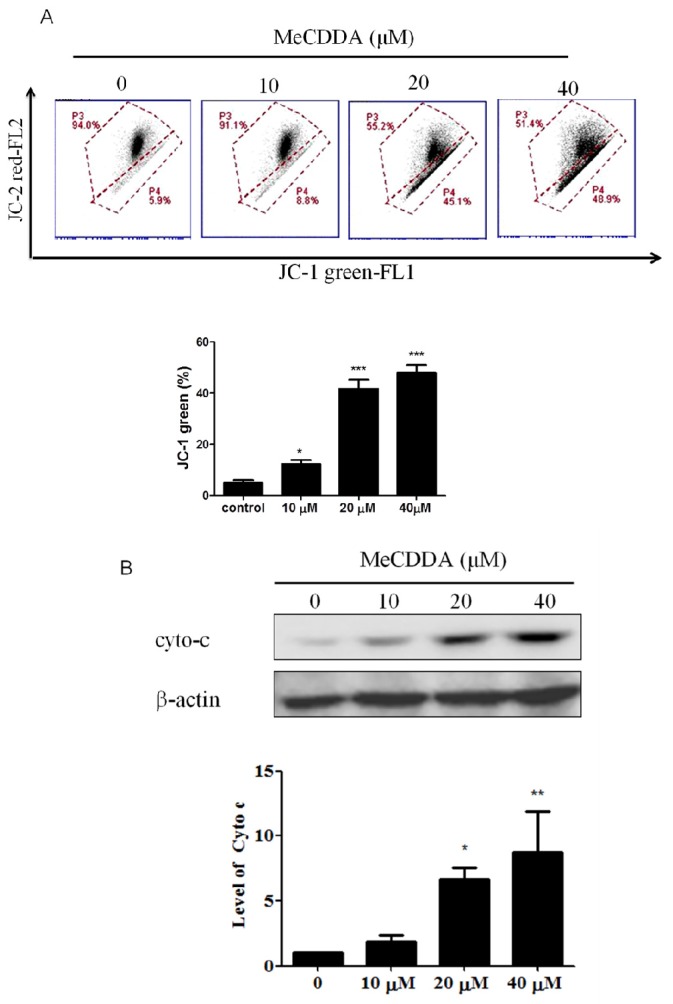
MeCDDA treatment caused impairment of mitochondrial membrane potential and an increase in cytochrome c release into the cytosol in H1688 cells. (**A**) H1688 cells were treated with MeCDDA or DMSO for 24 h. The cells were then stained with JC-1 fluorescence dye, and the mean JC-1 fluorescence intensity was determined using flow cytometry. (**B**) Cytosolic lysates were prepared and subjected to Western blotting. β-actin was using as a loading control. Densitometric analysis for protein expressions was performed using ImageJ software. The means ± SD of the triplicated experiments are presented in the bar graphs. * *p* < 0.05, ** *p* < 0.01, *** *p* < 0.001 compared with control (one-way ANOVA).

Loss of mitochondrial membrane potential (MMP) is an indicator of apoptosis [14]. Therefore, JC-1 fluorescence dye was used to evaluate the permeability of mitochondria membranes in H1688 cells treated with MeCDDA. As shown in Figure 4A, following treatment of MeCDDA, a dose-dependent decrease in the intensity of red fluorescence was observed in H1688 cells, suggesting that MeCDDA induced MMP reduction. A loss of MMP can lead to a release of cytochrome c from the mitochondria to the cytosol, a critical event during apoptosis [15]. Thus, the cytosolic fractions of H1688 cells treated with MeCDDA were extracted and cytochrome c release was determined by Western blotting analysis. As shown in Figure 4B, MeCDDA significantly induced an increase in cytochrome c expression in the cytosolic fraction of H1688 cells. Taken together, these data suggest that the mitochondrial pathway is responsible for the MeCDDA-induced apoptosis in H1688 cells.

### 2.5. MeCDDA Modulated Bcl-2 Family Protein Expression in H1688 Cells

**Figure 5:**
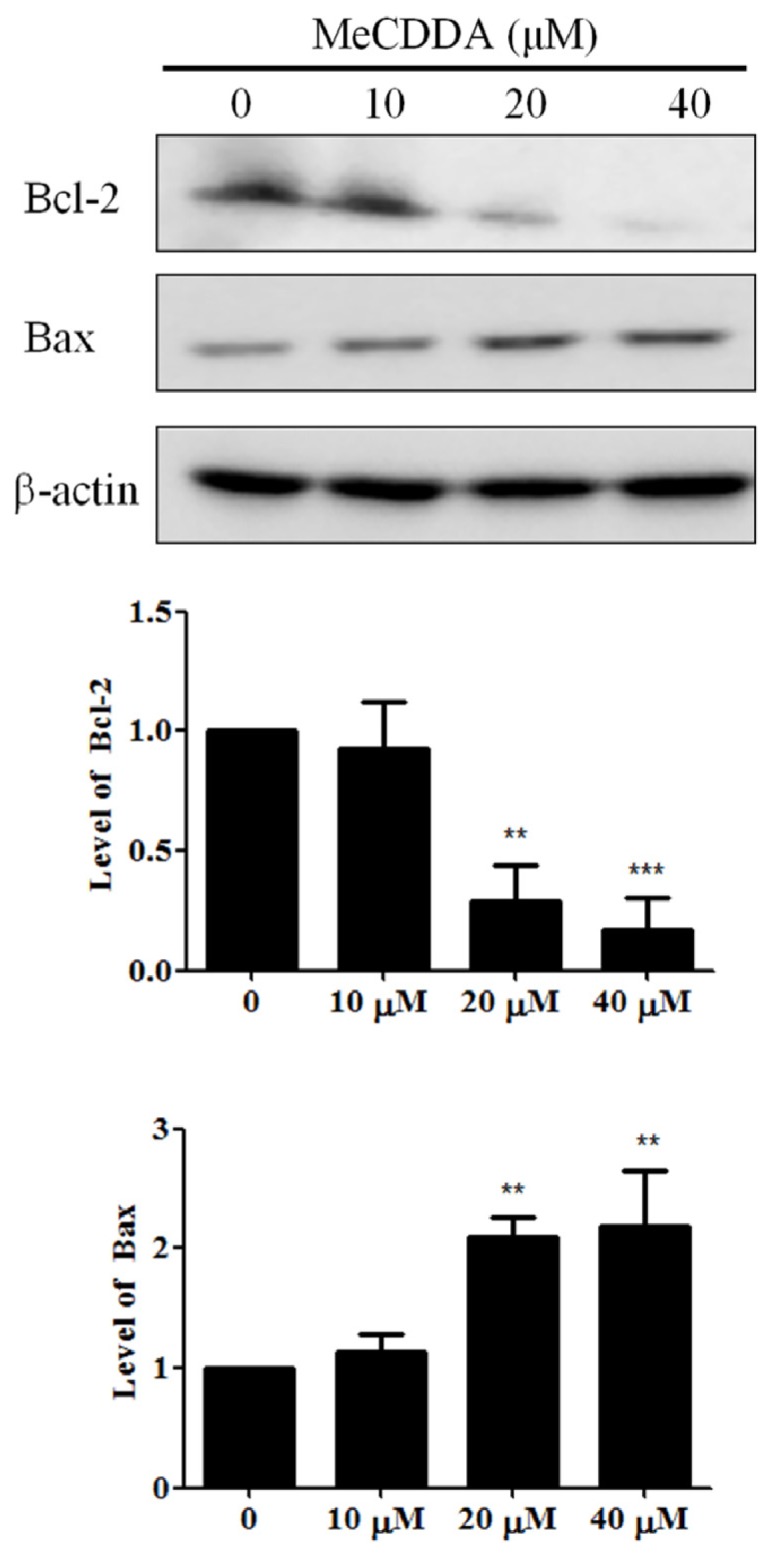
MeCDDA increased Bax and decreased Bcl-2 expression in H1688 cells. H1688 cells were treated with MeCDDA or DMSO for 24 h. Total lysates were prepared and subjected to Western blotting. β-actin was used as a loading control and the quantified expressions (mean ± SD) by Image J software were plotted in the bar graphs. ** *p* < 0.01, *** *p* < 0.001 compared with control (one-way ANOVA).

The Bcl-2 family proteins serve as a critical control point in the regulation of mitochondrial apoptosis by functioning as anti-apoptotic (Bcl-2) or pro-apoptotic (Bax) proteins in the cell death process [16]. Therefore, we analyzed the effects of MeCDDA on the expression of both Bax and Bcl-2 in H1688 cells. As shown in Figure 5, MeCDDA significantly increased Bax expression, but decreased Bcl-2 expression in H1688 cells.

### 2.6. MeCDDA Inactivated the PI3K/Akt Pathway in H1688 Cells

**Figure 6:**
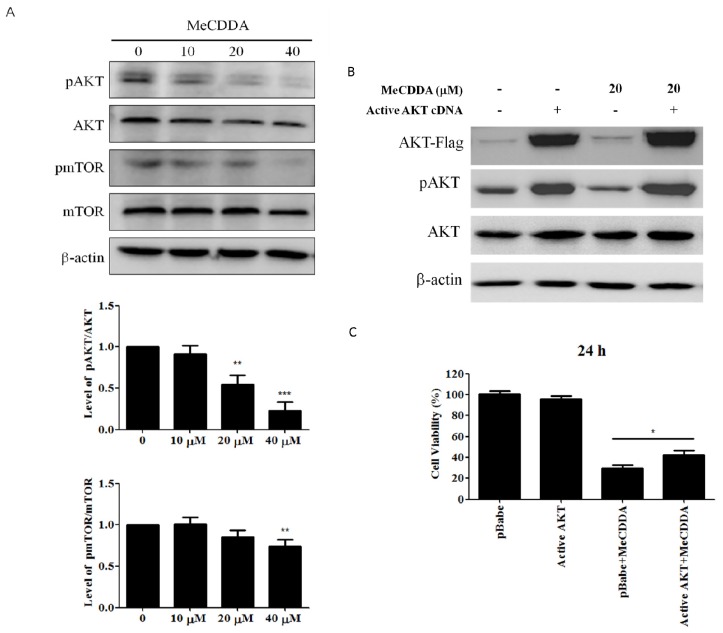
MeCDDA affected the PI3K/Akt/mTOR pathway in H1688 cells. H1688 cells were treated with MeCDDA or DMSO for 24 h. (**A**) Band image and the densitometric quantifications of Western blotting results. The bar graph shows the mean ± SD values from three independent experimental results. ** *p* < 0.01, *** *p* < 0.001 compared with control (One-Way ANOVA); (**B**) active AKT cDNA- or pBABE-transfected cells were treated with or without 20 µM MeCDDA for 24 h and subjected to Western blotting analysis using indicated antibodies; and (**C**) cell viability was evaluated at 24 h of treatment by MTT assay. Values in the bar graph are means ± SD from triplicated samples of each treatment, * *p* < 0.05 compared between pBABE + MeCDDA and AKT cDNA + MeCDDA groups (Student’s *t* test).

The PI3K/AKT/mTOR pathway is involved in a variety of cellular functions, including apoptosis [17]. Therefore, to investigate whether the effects of MeCDDA-induced apoptosis on H1688 cells are involved with this pathway, we assessed the effects of MeCDDA on phosphorylated-Akt (p-Akt) and phosphorylated-mTOR levels. The results shown in Figure 6A indicate that p-Akt and p-mTOR levels were significantly decreased in response to MeCDDA treatments, arguing the suppression of the Akt/mTOR pathway by MeCDDA could be responsible for the apoptosis of H1688 cells. To better elucidate the role of the PI3K/AKT/mTOR pathway in MeCDDA-induced apoptosis in lung cancer cells, we transfected an AKT cDNA which can constitutively express the AKT protein to H1688 cells and treated these AKT over-expressed cells with MeCDDA, measuring the cell viability to quantify the results. Our data in Figure 6B,C indicate overproduction of AKT in MeCDDA-treated cells attenuated the cytotoxicity induced by MeCDDA, suggesting that the PI3K/Akt/mTOR pathway seems to be utilized by MeCDDA in its pharmacological action.

### 2.7. MeCDDA-Inhibited H1688 Growth In Vivo

**Figure 7:**
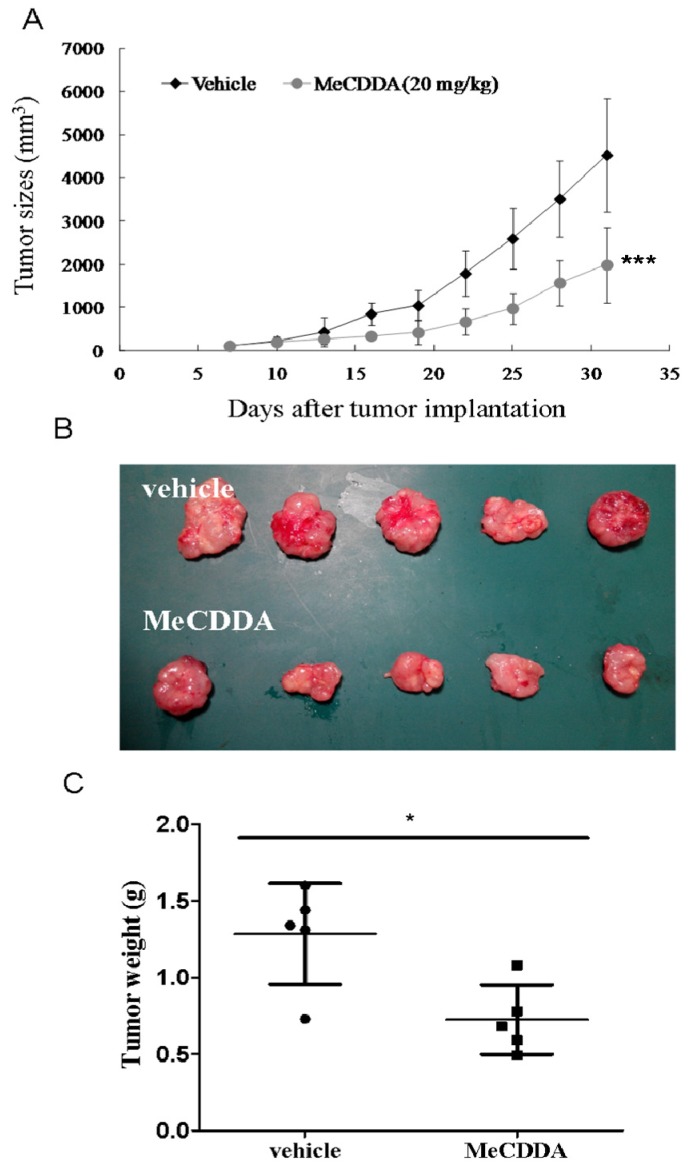
MeCDDA inhibited H1688 tumor growth in nude mice. H1688 cells (1 × 10^7^) were subcutaneously injected into the mice, allowing tumor cells to grow up to approximately 100 mm^3^ in size. MeCDDA (20 mg/kg) and vehicle control were administered intraperitoneally three times a week until Day 28 (*n* = 5). (**A**) The time course of tumor sizes. The photograph of tumor tissues (**B**) and the tumor weight (**C**) by Day 28 were demonstrated. Both quantified data are presented as means ± SD. * *p* < 0.05, *** *p* < 0.001 were the comparisons between vehicle and MeCDDA groups (two-way ANOVA for tumor size and Student’s *t* test for tumor weight).

We further examined whether MeCDDA could prevent H1688 tumor xenograft progression in nude mice. As shown in Figure 7A, the tumor volume in MeCDDA-treated mice at doses of 20 mg/kg was significantly reduced compared with vehicle controls (*** *p* < 0.001; two-way ANOVA). Notably, while MeCDDA may lead to some degree of harm to experimental mice, we observed that no mice exhibited obvious abnormal behaviors or died for non-tumor factors, suggesting the toxicity of MeCDDA could be low or very limited. Moreover, we measured the wet weights of the tumors and the results show that MeCDDA treatment significantly reduced tumor weight (* *p* < 0.05; Student’s *t* test; Figure 7B) compared with the vehicle-treated mice. These results indicate that MeCDDA also inhibits the growth of small lung cancer cells in vivo.

## 3. Discussion

Marine invertebrates have been shown to be a major source of bioactive compounds with anti-cancer activities [18,19]. In recent years, a variety of marine natural products within soft corals, have been found to be cytotoxic to various cancer cell lines [18,20,21,22]. Despite the reports on the potential anti-lung cancer activity of soft coral-derived metabolites, most are focused primarily on non-small cell lung cancer instead of small lung cancer, such as oxygenated steroids from *Nephthea chabrolii* and a dihydroaustrasulfone alcohol from *Cladiella australis*, exhibiting cytotoxicity towards A549 cells, the NSCLC cell line [23,24]. To the best of our knowledge, there are currently no studies on metabolites, namely steroids from soft coral, exhibiting apoptotic effects towards SCLC cells. Our findings in this study filled this niche of knowledge by demonstrating that MeCDDA isolated from *N. erecta* could induce apoptosis in H1688 cells in vitro and in a mouse xenograft model.

Though we have revealed that the anti-SCLC activity of MeCDDA could correlate to downregulation of PI3K/Akt/mTOR pathway (Figure 6A), the underlying mechanism might still require further thorough investigation. For example, clinical evidence has indicated that epidermal growth factor receptor (EGFR) mutation could be associated with SCLC in the non-smoking population [25,26]. Given EGF would activate the PI3K/Akt/mTOR signaling pathway [27], it is crucial to investigate whether or not MeCDDA can alter the expression level of EGFR on SCLC cells or induce conformational changes in EGF protein structure, achieving its anti-cancer effect. Other mechanisms of action could also be exerted by MeCDDA, such as microtubule rearrangement. Discodermolide isolated from a Caribbean marine sponge, *Discodermia dissolute*, has been shown to interfere with the stabilization of microtubulins within cancer cells by increasing polymerization [28,29]. As a result, we believe there are more mechanisms of action exerted by MeCDDA to be revealed.

We are also cognizant of the limitations of this study. Although the PI3K/Akt/mTOR pathway appears to be the mechanism of action of MeCDDA, its cytotoxic activity in H1688 cells was not as high as the synergistic effect from the combinatory administration of MeCDDA and LY294002, a PI3K/Akt inhibitor (data not shown). Furthermore, transfection of active AKT cDNA to H1688 cells did not return the cell viability back to the mock control levels despite the fact that the protein expressional level of phosphorylated AKT in transfected cells was similar to cell-only control (Figure 6B,C). This suggests that other signaling pathways, in addition to the PI3K/Akt/mTOR pathway, may contribute to the apoptosis activity of MeCDDA. Moreover, whereas we have demonstrated that MeCDDA can directly reduce the xenograft tumor volume caused by H1688, a spontaneous SCLC animal model would still be necessary. Currently, a compound conditional knockout of Rb and p53 mouse model (RPf mice) has been shown to develop mouse SCLC spontaneously [30]. Therefore, MeCDDA-induced reduction of xenograft tumor volume could be more conclusively demonstrated by treating RPf mice with MeCDDA. Regardless of the caveats existing in our study, we believe that our findings have opened up a wider aspect of soft coral-derived metabolites in chemotherapeutic applications.

Overall, our findings in this study have clearly demonstrated the potent effectiveness of MeCDDA from *Nephthea erecta* on SCLCs in vivo and expanded the possibility to apply such a steroid derivative in the treatment of tumors. Hence, we believe that potential pre-clinical candidate development could be driven by this study, leading to further investigation on marine bioactive compounds.

## 4. Materials and Methods

### 4.1. Chemicals

24-methyl-cholesta-5,24(28)-diene-3β,19-diol-7β-monoacetate (MeCDDA) was isolated from the wild-type soft coral *Nephthea erecta* as previously described [31] and kindly provided by Dr. Jui-Hsin Su (National Museum of Marine Biology and Aquarium, Pingtung, Taiwan). MeCDDA was dissolved in dimethyl sulfoxide (DMSO; Sigma-Aldrich, St. Louis, MO, USA) at 80 mM as a stock solution for in vitro assays. DMSO was used as the vehicle control.

### 4.2. Cell Lines

Human small cell lung cancer (SCLC) cell lines, H1688 and H146, were purchased from the Food Industry Research and Development Institute (Hsinchu, Taiwan) and grown in RPMI-1640 supplemented with 10% fetal bovine serum (FBS), 100 µg/mL penicillin, and 100 μg/mL streptomycin. The cells were incubated at 37 °C in a humidified atmosphere of 5% CO_2_.

### 4.3. Over-Expression of AKT Protein in Transfected H1688 Cells

pBabe-puro-Myr-Flag-AKT vector is a pBabe-puro-based plasmid. Both of the pBabe-puro and pBabe-puro-Myr-Flag-AKT vectors were obtained from Addgene (Cambridge, MA, USA). Briefly, 2.5 µg of each plasmid was transfected into HEK-293T cells to generate the retroviral particles, Moloney Murine Leukemia Virus, for 24 h followed by collection of viral particles from the medium and HEK-293T cells. 5 × 10^5^ of H1688 cells were cultured along with retrovirus and 8 µg/mL of polybrene (Sigma-Aldrich, St. Louis, MO, USA) for 48 h incubation, allowing H1688 cells to be transfected by the virus. Transfected cells were then subjected to the Western blotting analysis and MTT (3-(4,5-Dimethylthiazol-2-yl)-2,5-Diphenyltetrazolium Bromide) assays.

### 4.4. DNA Profile Analysis and Apoptosis Dectection

SCLC cells with or without MeCDDA treatments were collected and washed with PBS after 24-h treatment in complete medium. The cells were then fixed with 70% ice-cold ethanol at 4 °C overnight and stained with PI/RNase staining solution for 15 min in the dark for DNA profile analysis or using annexin V/PI binding kit for determining the percentages of apoptotic cells. An Accuri^TM^ C5 cytometer (BD Biosciences, San Jose, CA, USA) was used for both DNA profile analysis of SCLC cells and apoptosis detection.

### 4.5. Caspase-3, 8, and 9 Activities Assay

After treatment with MeCDDA for 24 h, cells were collected for measurement of caspase-3, 8, and 9 activities using the appropriate CaspGLOW™ Fluorescein Active Caspase Staining Kits (Biovision, Milpitas, CA, USA) according to the manufacturer’s specifications.

### 4.6. Mitochondrial Membrane Potential Assay

Mitochondrial membrane potential was analyzed using the fluorescent potentiometric dye JC-1 according to the manufacturer’s instructions. Briefly, after cells were treated with MeCDDA for 24 h, the cells were pelleted, washed with PBS, and stained with 10 µg/mL JC-1 (Invitrogen™; Thermo Fisher Scientific, Inc., Waltham, MA, USA) for 20 min. Subsequently, the stained cells were qualitatively analyzed with an Accuri^TM^ C5 cytometer.

### 4.7. Cytochrome C Measurement

A mitochondria isolation kit for cultured cells (Thermo Fisher Scientific, Inc., Waltham, MA, USA) was used for cytochrome c analysis, according to the manufacturer’s protocol. Briefly, cells were treated with MeCDDA for 24 h, and then resuspended in 800 µL of Reagent A on ice for 2 min. Following incubation, 10 µL Reagent B was added, and cells were incubated on ice for 5 min and vortexed each minute. Subsequently, 800 µL Reagent C was added, cells were centrifuged at 700× *g* for 10 min at 4 °C, and the resulting supernatant was centrifuged at 10,000× *g* for 10 min. The supernatant was collected as cytosolic extracts for the analysis of cytochrome c. Concentration of cytochrome c in cytosolic extracts was determined by Western blotting and normalized to β-tubulin.

### 4.8. Western Blotting Analysis

Cells were treated with MeCDDA in complete medium for 24 h. Total protein was isolated using RIPA lysis buffer, and the protein concentrations of lysates were determined using the BCA Protein Assay Kit. Equal amounts of each protein were separated on sodium dodecyl sulfate (SDS)–polyacrylamide gel electrophoresis and transferred to a polyvinylidene fluoride membrane. Membranes were then immersed in a blocking solution (Tris-buffered saline with Tween-20 (0.01% Tween), and 5% non-fat milk), and the blot was incubated with primary antibodies overnight at 4 °C, followed by a 1-h incubation with horseradish peroxidase–conjugated secondary antibodies. Membranes were developed using the enhanced chemiluminescence detection kit reagent (ECL prime western blotting Substrate, GE Healthcare Life Sciences, Piscataway, NJ, USA) to detect specific proteins. The primary antibodies to Bax, Bcl-2, cytochrome c, p-AKT, AKT, p-mTOR, mTOR, Flag, and β-actin were purchased from Cell Signaling Technology (Danvers, MA, USA). All the densitometric analysis performed in this study was conducted using ImageJ software (National Institute of Health, Bethesda, MD, USA).

### 4.9. Animals

Male BALB/c (Bagg albino, laboratory-bred strain of the House Mouse) six-week-old nude mice (nu/nu) (20 ± 2 g) were obtained from the National Laboratory Animal Center (Taipei, Taiwan) and raised in the National Chung Hsing University (NCHU) Animal Experiment Research Center. The mice were fed sterilized mouse chow and water. Laboratory animal management was overseen by the Laboratory Animal Management and Ethics Committee of NCHU.

### 4.10. MeCDDA Treatment of Xenograft Tumors

H1688 cells (1 × 10^7^) were suspended in 0.2 mL of extracellular matrix gel (BD, Biosciences Discovery Labware, Bedford, MA, USA) and injected into the back of the animal under the skin. When the tumors were established (day 10), the mice were randomly divided into two groups: the vehicle control group was given 10% DMSO + 90% glyceryl trioctanoate (Sigma-Aldrich, St. Louis, MO, USA), while the MeCDDA treatment group received 20 mg/kg of MeCDDA via intraperitoneal injection (i.p.) three times a week. The tumor parameters were measured three times per week with an electronic caliper, and the tumor volume was calculated using the formula for tumor volume (mm^3^) = a × b^2^ × 0.5, where a is the length of the tumor, and b is the width of the tumor. Mice were sacrificed 28 days after the start of treatment. All tumors excised from the mice were weighed.

### 4.11. Statistical Analysis

The data were analyzed with GraphPad Prism Version 5.0 (San Diego, CA, USA) and values of * *p* < 0.05 were considered statistically significant. For comparison between two groups, we used unpaired two-tailed *t* test (Student’s *t* test). One-way or two-way ANOVA was used to compare multiple groups according to the experiments. All values are expressed as the mean ± SD.

## Figures and Tables

**Table 1:** DNA profile analysis of H1688 cells treated with MeCDDA.

Group	Sub-G_1_	G_0_/G_1_	S	G_2_/M
Control	0.9 ± 0.2	62.0 ± 0.4	16.8 ± 0.4	19.9 ± 0.2
MeCDDA 10 μM	1.2 ± 0.2	62.0 ± 1.2	17.1 ± 0.6	20.4 ± 0.4
MeCDDA 20 μM	32.5 ± 2.7 ***	39.4 ± 1.4 ***	12.8 ± 1.6 *	15.0 ± 1.6 *
MeCDDA 40 μM	89.9 ± 5.8 ***	4.9 ± 0.4 ***	2.7 ± 2.4 ***	2.8 ± 3.1 *

Results are expressed as mean % of cells in a specific phase of cell cycle ± SD. * *p* < 0.05, *** *p* < 0.001 indicate significant difference compared to the control group (one-way ANOVA).
